# Early results of transcatheter electrosurgical aortic septotomy for endovascular repair of chronic dissecting aortoiliac aneurysms

**DOI:** 10.1016/j.jvscit.2024.101467

**Published:** 2024-02-29

**Authors:** Mira T. Tanenbaum, Andres V. Figueroa, K. Benjamin Lee, Jose Eduardo Costa Filho, Marilisa Soto Gonzalez, Mirza S. Baig, Carlos H. Timaran

**Affiliations:** Division of Vascular and Endovascular Surgery, Department of Surgery, University of Texas Southwestern Medical Center, Dallas, TX

**Keywords:** Aortic aneurysm, Aortic septotomy, Chronic aortic dissection, Endovascular aortic repair, Transcatheter electrosurgery

## Abstract

**Objective:**

Endovascular repair of chronic dissecting aortoiliac aneurysms is challenging given the rigid septum, compressed true lumen (TL), and target vessels frequently originating in the false lumen. We have used transcatheter electrosurgical aortic septotomy (TEAS) before stent graft implantation under intravascular ultrasound (IVUS) and fusion guidance. The purpose of this study is to assess the outcomes of TEAS during complex endovascular repair of dissecting aneurysms.

**Methods:**

From 2021 to 2023, 17 patients underwent TEAS. The primary end point was technical success, with secondary end points of proximal and distal seals, target vessel instability, aortic and iliac TL and cross-sectional area (CSA) expansion, and aortic-related death. During the procedure, the aortic septum is crossed through a pre-existing entry or via electrocautery-activated 0.018-in. Astato XS20 wire (Asahi-Intecc) under IVUS and fusion guidance. The penetrated wire is then snared in the false lumen and pulled through the ipsilateral femoral access. A 1-cm length of the middle of the Astato wire coating is kinked in a three-sided polygonal configuration, denuded the inner surface of the wire using a no. 15 blade, and positioned at the apex of the septum. Both ends of the Astato wire are insulated with 0.018-in. microcatheters, and the back end of the wire is denuded and connected to cautery. Gentle traction is applied to the wire, and short bursts of electrocautery cutting are applied at 60 to 80 W.

**Results:**

The technical success of the septotomy was 100%. No incidence of visceral or lower extremity malperfusion, vascular injury, or distal embolization occurred. Of the 17 patients, 4 underwent thoracic endovascular aneurysm repair, 2 underwent endovascular aortic repair, and 11 underwent fenestrated/branched endovascular aneurysm repair after septotomy. All target vessels were successfully stented. A distal landing zone seal with exclusion of the false lumen was achieved in 16 of the 17 patients (94.1%). One patient required embolization of the false lumen of the celiac artery after septotomy. The TL mean diameter and CSA of the descending thoracic aorta after septotomy was expanded by 7.01 ± 1.9 mm (relative mean diameter expansion, 42.3%; *P* < .0001) and 2.71 ± 0.4 cm^2^ (relative mean CSA expansion, 57.3%, *P*<.0001). For patients who required septotomy through the common iliac arteries, the mean TL was expanded by 8.1 ± 3.7 mm (relative mean diameter expansion, 76%; *P* < .0001) and 1.76 ± 0.91 cm^2^ (relative mean CSA expansion, 209%; *P* < .0001). The 1-year freedom from target vessel instability was 91%.

**Conclusions:**

The use of IVUS and fusion-guided TEAS offers a promising technique to facilitate TL expansion and false lumen exclusion in chronic dissecting aortic aneurysms before repair. The durability and long-term outcomes of this technique in a larger cohort remain to be elucidated.

**Article Highlights**

• **Type of Research:** A single-center, retrospective cohort study
• **Key Findings:** Intravascular ultrasound- and fusion-guided transcatheter electrosurgical aortic septotomy (TEAS) is safe for patients with chronic dissecting aortoiliac aneurysms and allows for great true lumen expansion. The use of TEAS before complex endovascular aortic repair facilitates proximal and distal landing zone seals with exclusion of the false lumen, without causing embolic adverse events or compromising target vessel cannulation, stenting, or stability.
• **Take Home Message:** TEAS provides a novel technique to address chronic dissecting aortoiliac aneurysms before complex endovascular aneurysm repair.

Chronic dissecting aortoiliac aneurysms represent a complex and challenging pathology encountered in vascular surgery. These aneurysms are characterized by progressive dilatation of the aortic and iliac segments after prior aortic dissection. Historically, open surgical techniques have been the cornerstone of treatment for these aneurysms, providing durability but carrying major risks inherent to thoracoabdominal surgery.^1^ With the evolution of endovascular technologies, minimally invasive options have emerged as alternatives in select patients.^2^

Endovascular repair of chronic dissecting thoracoabdominal aortoiliac aneurysms (TAAAs) has achieved increasing attention, given its potential to mitigate the morbidity associated with open surgical interventions.^3^ Endovascular approaches focus on entry tear coverage and discontinuation of blood entry into the false lumen to prevent progression of disease. In chronic dissection, endovascular repair remains challenging given the septum rigidity, compression of the true lumen (TL), and target vessels arising from the false lumen. Existing techniques, such as cheese wire fenestration,^4^^,^^5^ balloon dissection flap fracture,^6^ and focused laser fenestration,^7^ aim to either obliterate the false lumen or create a common aortic lumen, which will allow for improved endovascular aortic repair (EVAR) device proximal and distal seals and visceral branch perfusion.^8^

Transcatheter electrosurgery has emerged as a field of procedures using radiofrequency energy, delivered through a catheter-based system, to create targeted and controlled thermal effects within the vascular lumen to vaporize and traverse or lacerate tissue under fluoroscopic or echocardiographic guidance.^9^ Procedures such as the BASILICA (bioprosthetic or native aortic scallop intentional laceration to prevent iatrogenic coronary artery obstruction), LAMPOON (laceration of the anterior mitral leaflet to prevent left ventricular outflow obstruction), and ELASTIC (electrosurgical laceration of Alfieri stitch prior to transcatheter mitral valve replacement) procedures use cutting radiofrequency concentrated on a target medium to induce vaporization.^10^, ^11^, ^12^, ^13^ In the context of chronic dissecting aortoiliac aneurysms, transcatheter electrosurgery offers distinct potential benefits, including endovascular exclusion of the false lumen, restoration of the TL, and creation of safe landing zones for EVAR to reinforce the weakened aortic wall. We used transcatheter electrosurgical aortic septotomy (TEAS) before EVAR with stent graft implantation under intravascular ultrasound (IVUS) and fusion guidance to divide the septum in its entirety via vaporization. This study aims to assess the safety and outcomes of this novel surgical technique.

## Methods

### Study cohort selection

A retrospective review was performed of a prospective database of consecutive patients undergoing endovascular aneurysm repair for aortic pathology during a 2-year period as part of a physician-sponsored investigational device exemption study at a single institution. The institutional review board approved the present study. The patients provided written informed consent to undergo repair with investigational devices (IDE no. G140108; NCT no. 02266719). All the patients in the series underwent EVAR for chronic dissecting aortoiliac aneurysms following TEAS. Demographic information, imaging studies, procedural details, postoperative outcomes, and follow-up data were collected. Technical success was defined using the Society for Vascular Surgery reporting standards regarding completion of the septotomy and delivery of the aortic and all side brand components for EVAR, thoracic EVAR (TEVAR), or fenestrated/branched EVAR (FBEVAR), and successful aneurysm sac exclusion.^14^ The aneurysm, TL size, false lumen size, and aneurysm characteristics were determined using preoperative and postoperative thin-slice computed tomography angiography (CTA) evaluated on a three-dimensional workstation (TeraRecon).

### End points

The primary end point was technical success, defined as successful septotomy with no evidence of visceral or lower extremity malperfusion or vascular injury. The primary end point of the study specifically assessed the aortic and common iliac TL diameter and cross-sectional area (CSA) expansion. The secondary end points included the proximal and distal seals, target vessel instability, aortic and iliac TL and CSA expansion, distal embolic events, early and late morbidity, mortality, and aortic-related death. Early outcomes were analyzed and defined as occurring during the hospital stay or ≤30 days after septotomy. The presence of endoleaks, access-related complications, need for secondary endovascular interventions, open surgical repairs, or medical interventions were included in the study.

### TEAS technique

**Fig 1:**
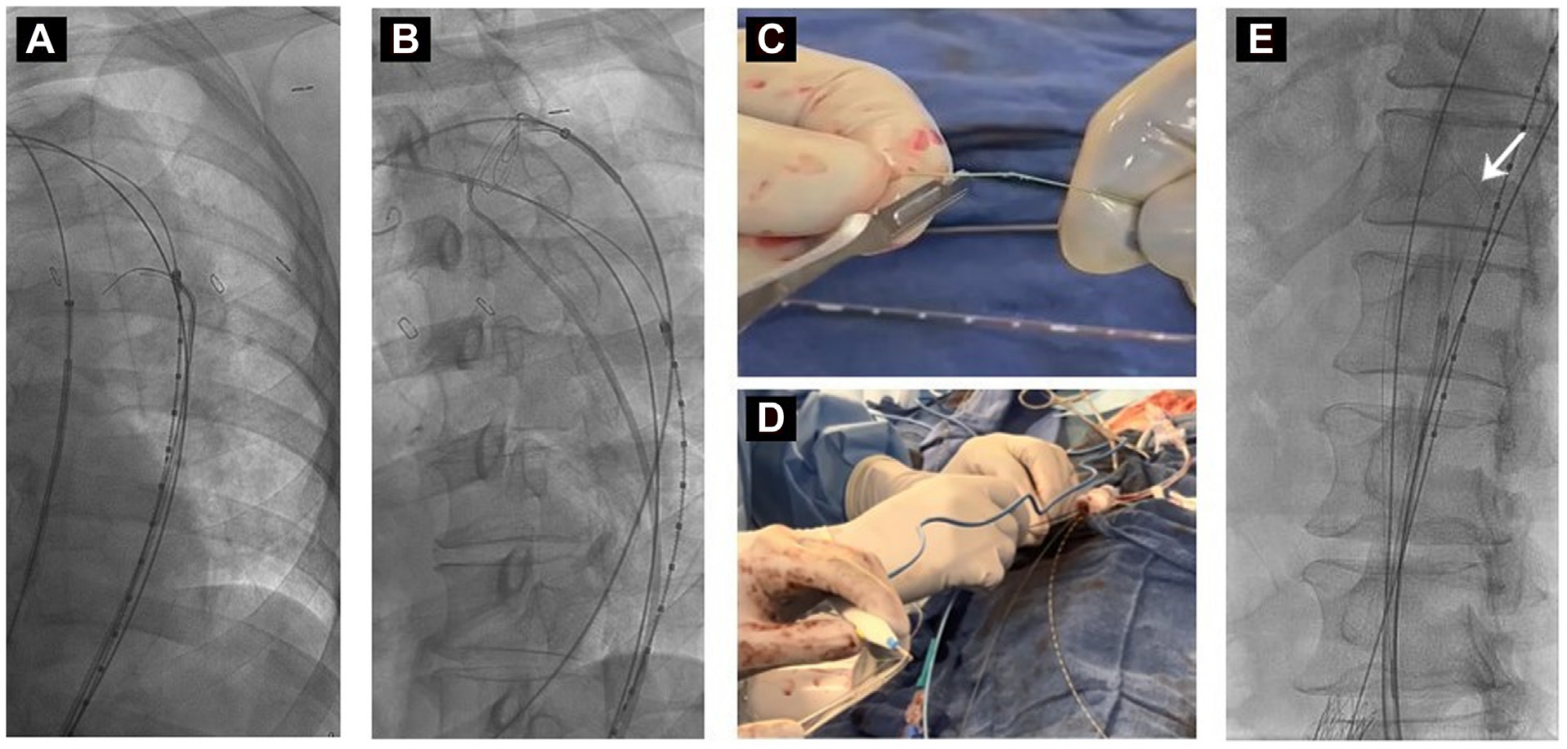
Demonstration of transcatheter electrosurgical aortic septotomy (TEAS) procedure technique with crossing of the septum with a 0.018-in. Astato XS20 wire (**A**) and snared via contralateral femoral access (**B**). The Astato wire is kinked, denuded (**C**), and connected to Bovie electrocautery (**D**). The kinked portion of the wire is positioned at the septum apex (white arow), with septotomy performed using gentle traction via ipsilateral femoral access and bursts of cutting electrocautery (**E**).

Bilateral femoral access was established under ultrasound guidance. Percutaneous closure devices were used as indicated. IVUS was then performed to evaluate the aorta, visceral vessels, and bilateral common iliac arteries. Unilateral groin access was upsized to an 18F sheath (DrySeal; W.L. Gore & Associates). Heparinization was performed with the goal of an activated clotting time >300 seconds, as is standard for complex endovascular aortic procedures. One Astato XS20 wire (Asahi-Intecc) was inserted into the TL. Another Astato wire was inserted into the false lumen, which was usually accessed under IVUS and fusion guidance through a distal entry site in the proximal external iliac or distal common iliac arteries. From the contralateral femoral access, the IVUS catheter was advanced into the TL. Using an Aptus TourGuide Steerable Sheath (Aptus Endosystems) or an internal mammary guiding catheter (Cordis), the septum was crossed from the TL into the false lumen (Fig 1, *A*) through a preexisting entry or via electrocautery-activated 0.018-in. Astato XS20 wire under IVUS and fusion guidance. The penetrating wire was snared using the Indy OTW vascular retriever (Cook Medical) in the false lumen (Fig 1, *B*) and pulled through the ipsilateral femoral access.

On a back table, a 1-cm length of the inner surface of the midportion of the Astato wire coating was denuded using a no. 15 blade and kinked in a three-sided polygonal configuration (Fig 1, *C*). This process requires 1 to 2 minutes to perform. Each end of the Astato wire was insulated with 0.018-in. microcatheters up to the bent, denuded portion. The back end of the Astato wire was also denuded and connected to a Bovie cautery (Fig 1, *D*). The kinked portion of the Astato wire was positioned at the apex of the septum (Fig 1, *E*). Short bursts of electrocautery were applied in cutting mode at 60 to 80 W, and gentle traction was applied to the wire as the septum was transected. If any resistance was encountered, the traction was discontinued. The length of septotomy required was determined from the preoperative CTA with the goal of dividing the membrane enough to allow for both proximal and distal landing zone seals and expansion of the visceral segment. Postseptotomy IVUS and angiography were performed to ensure aortic and visceral vessel patency with blood flow via the TL. IVUS immediately after TEAS also allowed for assessment of the edges of the septum, confirming appropriate septotomy and stability of the retracted portion of the septum. No further interventions were required to extend the septotomy or further expand the divided membrane.

The stent grafts were deployed as indicated. Staged procedures usually included TEVAR and/or iliac stenting. Nonstaged procedures involved FBEVAR after TEAS. Postoperatively, patients maintained strict bed rest for ≥6 hours to prevent access site complications. Ambulation and antiplatelet therapy usually began on postoperative day 1. All patients were discharged with low-dose aspirin therapy. Depending on the comorbidities and/or presence of small or diseased target vessels, some patients were discharged with dual-antiplatelet therapy for the first 3 months and reevaluated on follow-up. Patients were scheduled for follow-up within 30 days with CTA at that time.

### Statistical analysis

Paired *t* tests were used to analyze the pre- and postseptotomy TL diameter and CSA measurements for each patient. The measurements were assessed for normal distribution, with a *P* value of < .05 considered statistically significant. Target vessel instability was defined as any branch-related death, rupture, occlusion, or reintervention for stenosis, kink, endoleak, or disconnection. The time-dependent outcome was reported using Kaplan-Meier estimates analysis. All analyses were performed using SAS, version 9.4 (SAS Institute Inc).

## Results

### Demographic data

A total of 17 patients underwent TEAS for a dissecting aortic aneurysm before EVAR between 2021 and 2023. Of the 17 patients, 7 (42%) had a type A dissection and 10 (58%) had a type B dissection. The mean age was 65 ± 9.5 years, and 15 patients (88%) were men. The most frequent comorbidities at baseline were hypertension in 17 patients (100%), hyperlipidemia in 15 (88%), smoking in 13 (76%), coronary artery disease in 8 (47%), and arrhythmia in 7 (42%). The patient demographics and comorbidities are listed in Table I. The mean aneurysm diameter was 59 ± 8.5 mm. Of the 17 patients, 2 (12%) were treated with EVAR, 4 (23%) with TEVAR, and 11 (65%) with FBEVAR after septotomy. Of the 11 patients treated with FBEVAR, the aneurysm anatomic location included one type III TAAA (9%), two type I TAAAs (18%), and eight type II TAAAs (72%). A total of 44 target vessels were stented, using 12 fenestrations (27%) and 32 branches (73%). Of the 44 target vessels, 12 (27%) arose from the false lumen, with the left renal artery the most frequent target vessel arising from the false lumen (66%).

**Table I:** Baseline patient demographics and clinical characteristics (n = 17)

Variable	Median (IQR) or No. (%)
Age, years	64.8 (62.5-67.1)
Sex	
Male	15 (88.2)
Female	2 (11.8)
Race	
White	8 (47.1)
Black	7 (41.2)
Postseptotomy procedure	
TEVAR	4 (23.5)
EVAR	2 (11.8)
FBEVAR	11 (64.7)
None	0 (0)
Dissection type A	7 (41.2)
Comorbidities	
HTN	17 (100)
CAD	8 (47.1)
Prior MI	2 (11.8)
ESRD	1 (5.9)
DM	2 (11.8)
Smoking history	13 (76.5)
BMI, kg/m^2^	30.7 (27.8-32.9)
ASA class >3	7 (41.2)
Maximum aortic diameter, mm	58.0 (52.0-61.5)

*ASA*, American Society of Anesthesiologists; *BMI*, body mass index; *CAD*, coronary artery disease; *DM*, diabetes mellitus; *ESRD*, end-stage renal disease; *EVAR*, endovascular aortic repair; *FBEVAR*, fenestrated/branched endovascular aortic repair; *HTN*, hypertension; *IQR*, interquartile range; *MI*, myocardial infarction; *TEVAR*, thoracic endovascular aortic repair.

### Technical success

**Fig 2:**
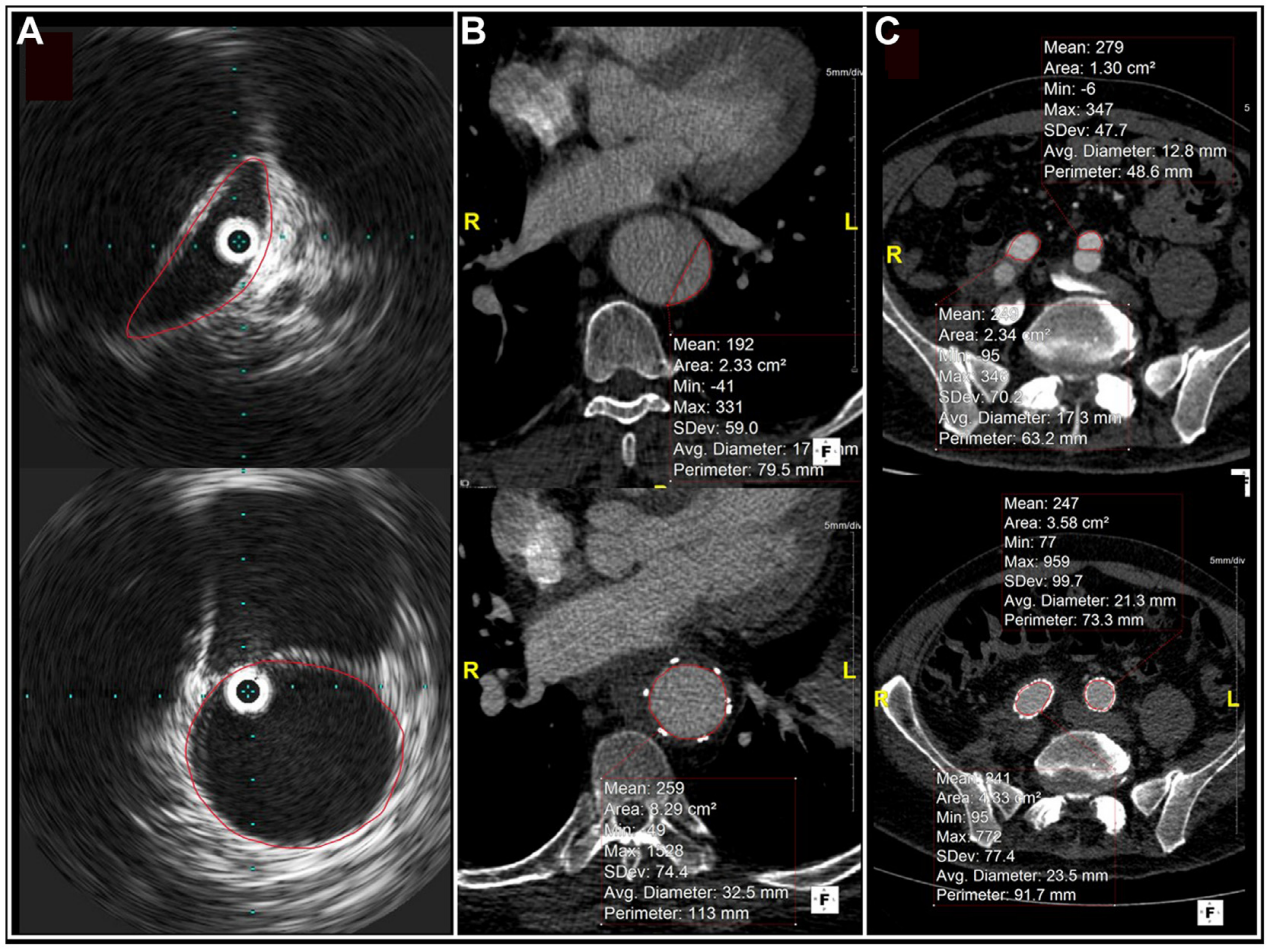
Representative comparative images from intravascular ultrasound (IVUS; **A**) and computed tomography angiography (CTA) of the aorta (**B**) and common iliac arteries (**C**) before and after transcatheter electrosurgical aortic septotomy (TEAS).

**Fig 3:**
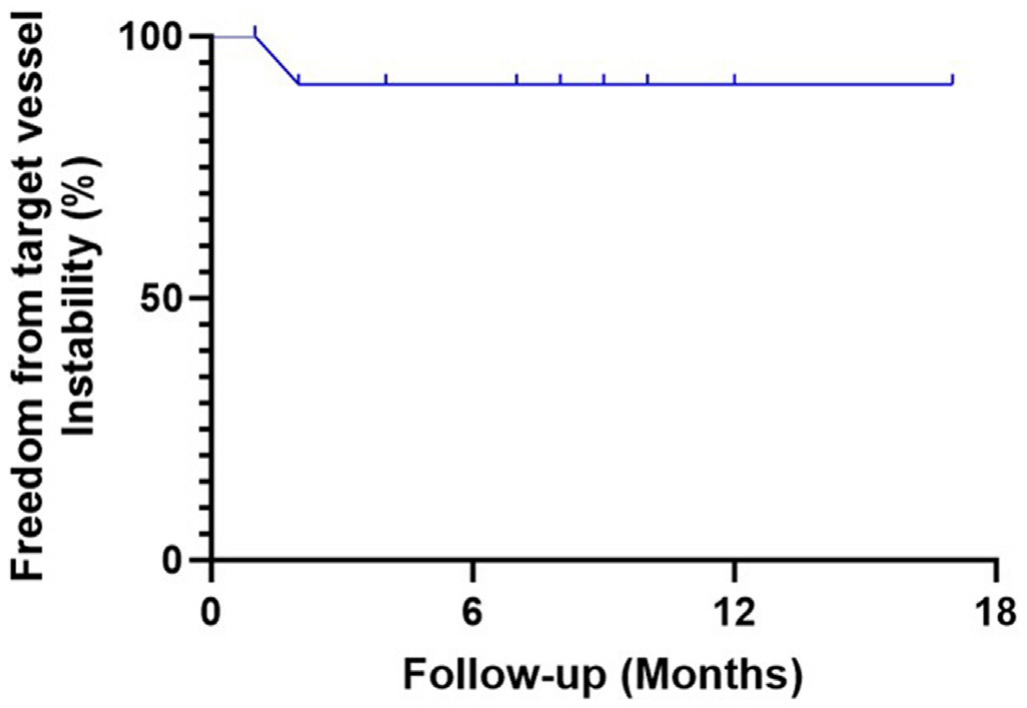
Kaplan-Meier estimates of any target vessel instability in 17 patients treated with transcatheter electrosurgical aortic septotomy (TEAS) for dissecting thoracoabdominal aortic aneurysms (TAAAs).

TEAS technical success was 100%. No difficulties related to the divided dissection flap were appreciated during the procedure. IVUS was used in all cases (Fig 2, *A*) before and after TEAS to confirm expansion of the TL and retraction of the divided septum. The operative indications and perioperative parameters are listed in Table II. No case of visceral or lower extremity malperfusion, vascular injury, or distal embolization occurred. All target vessels during FBEVAR were successfully stented. Upper extremity access was used in eight patients (47%). Proximal and distal landing zone seals with the exclusion of false lumen were achieved in 16 of the 17 patients (94.1%). The aortic TL mean diameter was expanded by 7.01 ± 1.9 mm, with a relative expansion of 42.3% (*P* < .0001). The aortic CSA expanded by 2.71 ± 0.4 cm^2^, with a relative expansion of 57.3% (*P* < .0001; Fig 2, *B*). Ten patients (58.8%) underwent septotomy extending to one or both common iliac arteries, with four of these patients (23.5%) requiring bilateral iliac artery septotomy. The technical success of iliac septotomy was 100%, without any occurrence of iliac artery injury or distal limb ischemia. The common iliac TL mean diameter was expanded by 8.1 ± 3.7 mm, with a relative expansion of 76% (*P* < .0001). The common iliac CSA was expanded by 1.76 ± 0.91 cm^2^, with a relative expansion of 209% (*P* < .0001; Fig 2, *C*). A total of five patients (29.4%) required secondary interventions. One patient required embolization of the false lumen of the celiac artery after septotomy. One patient was found to have a pseudoaneurysm at the right common femoral access site, which, after multiple failed attempts at thrombin injection, required open repair. Four patients (23.5%) had type II endoleaks, with only one requiring embolization of an iliolumbar artery branch. One patient (5.88%) had a type Ia endoleak, which was successfully treated with proximal extension using a TEVAR cuff, and a type Ib endoleak, which was treated with embolization using three Amplatzer vascular plugs and distal extension of the common iliac artery stent. At a mean follow-up of 6.3 ± 1.2 months, the primary patency was 100%. The estimated 1-year freedom from target vessel instability was 91% (Fig 3).

**Table II:** Operative indications and perioperative parameters (n = 17)

Variable	No. (%) or median (IQR)
Aneurysm classification	
Descending thoracic aneurysm	4 (23.5)
AAA	2 (5.9)
TAAA	11 (64.7)
Type I	2 (11.8)
Type II	8 (47.1)
Type III	1 (5.9)
Type IV	0 (0)
Dissection classification	
A	7 (41.2)
B	10 (58.8)
Definitive EVAR device	
TEVAR	4 (23.5)
EVAR	2 (11.8)
FBEVAR	11 (64.7)
Staged procedures	2 (5.9)
Aortic TL diameter, mm	
Before TEAS	16.2 (12-18.5)
After TEAS	23.3 (20.0-26.9)
Aortic CSA, cm^2^	
Before TEAS	205 (118.0-261.5)
After TEAS	428 (280.8-583.8)
Iliac TL diameter, mm	
Before TEAS	10.4 (10.7-12.7)
After TEAS	18.8 (17.1-22.2)
Iliac CSA, cm^2^	
Before TEAS	73.5 (69.9-105.0)
After TEAS	238.5 (239.0-357.0)
Procedure parameters	
Operative time, minutes	275 (206-369)
Fluoroscopy time, minutes	90.9 (56.5-112.7)
DAP, Gy∗cm^2^	267 (136-386)
RAK, mGy	1352.21 (927.3-2597.2)
EBL, mL	200 (150-250)

*AAA*, Abdominal aortic aneurysm; *CSA*, cross-sectional area; *DAP*, dose area product; *EBL*, estimated blood loss; *EVAR*, endovascular aortic repair; *FBEVAR*, fenestrated/branched endovascular aortic repair; *IQR*, interquartile range; *RAK*, reference air kerma; *TAAA*, thoracoabdominal aortic aneurysm; *TEAS*, transcatheter electrosurgical aortic septotomy; *TEVAR*, thoracic endovascular aortic repair; *TL*, true lumen.

## Discussion

The results of this study show that IVUS- and fusion-guided TEAS can be used safely and effectively for patients with dissecting TAAAs. Our study revealed that TEAS offers great TL and CSA expansion, which allows for the deployment of larger devices and improves device maneuverability during the procedure. Furthermore, the creation of a common lumen using TEAS facilitates cannulation of target vessels arising from the false lumen, allowing for preservation of perfusion to the visceral organs. Specifically, fusion guidance helps ensure that the Astato wire is appropriately positioned at the apex of the septum before starting TEAS. Furthermore, during TEAS, visualization of the Astato wire on the fusion-guided image helps to limit wire movement that might encroach on the aortic walls. The use of IVUS during these procedures, not only aids in enhancing fusion registration and guiding the septotomy itself, but also provides an immediate evaluation of the newly created common aortic lumen and visceral vessel perfusion. The immediate visualization of the now divided and retracted septum aids the operators in confirming adequate septum vaporization and ensuring that the divided dissection flap does not occlude any visceral vessel ostia. Previous data report a freedom from target vessel instability at 1 year after FBEVAR of 91%, comparable to that found in this study.^15^

One unique aspect of this procedure is conformation of the Astato wire used to perform TEAS. Reviews of established transcatheter electrosurgical techniques, mainly used in interventional cardiology, describe using a focus of the energy at either a wire tip or the apex of a “flying V” configuration of a guidewire, such as seen in the BASILICA, LAMPOON, and ELASTIC procedures.10,11,12 Specifically, the “flying V” configuration can cut tissue by concentrating the charge at the inner lacerating surface of the guidewire and eliminating alternative current paths using a dextrose flush during electrocauterization.^9^ These techniques are also seen in previously described surgical approaches addressing dissection flaps in dissecting abdominal aortic aneurysms. These include laser fenestration using a focused energized tip and the cheese wire septotomy technique using a V-shaped wire with manual traction to divide the dissection flap.^4^^,^^5^ TEAS uses a polygonal wire configuration, which still focuses most of the energy at the septum, but also allows for a slightly wider concentration of energy. This theoretically addresses the thickening of the septum and associated clot seen in chronic dissecting aortoiliac aneurysms without allowing for alternative current paths to cause collateral damage to surrounding tissue.

In the traditional cheese wire septotomy technique, the septum is divided by mechanical traction of the V-shaped wire. In contrast, TEAS is a purely electrosurgical technique that relies on focused radiofrequency energy applied to the septum to divide it via focal vaporization of the thickened septum. Very gentle traction is applied while the electrosurgical energy is delivered. Avulsion of the septum with the resulting aortic, iliac, or target artery occlusion frequently seen with the mechanical cheese wire technique was not seen in this series, because local trauma to the aorta and septum is minimal with TEAS. In contrast to electrocautery, the use of electrosurgical septotomy to vaporize the target tissue as described in this study minimizes the risk of intravascular coagulation or possible thromboembolism and also limits the charring of surrounding tissue.^13^ Furthermore, the use of fusion guidance during TEAS helps to ensure that the Astato wire remains in an optimal position to prevent collateral damage to the aortic or iliac walls.

Previously described electrosurgical techniques use a lower level of wattage during the procedure. The BASILICA procedure uses 30 W of electrocautery cutting to effectively lacerate the aortic leaflet and limit excessive tissue damage and possible generation of debris or emboli. ELASTIC is another electrosurgical technique used before transcatheter mitral valve replacement. Compared with the BASILICA and LAMPOON techniques, it uses a higher level of radiofrequency energy application at 70 W with a continuous dextrose infusion.^10^, ^11^, ^12^ Prior literature suggests that the voltage needed to traverse tissue is 70 V, which is achieved when 5 to 13 W of power is applied, with variance based on the wire and insulation used.^13^^,^^16^ The higher level of wattage during the ELASTIC procedure is chosen because electrosurgical laceration requires significantly more energy than just tissue traversal.^12^ Using these guiding principles, we were able to select a radiofrequency energy application of 60 to 80 W to effectively vaporize the thickened chronic septum without any identified collateral damage in this study. Another potential advantage of using higher energy is the retraction of the septum observed in all our cases after TEAS, which resulted in wider communication between the TL and false lumen and stability of the septum edges, preventing target vessel occlusion.

In the initial cases, we did not use a dextrose flush during TEAS. Currently, we are using an influx of dextrose through the steerable sheath or guiding catheter to focus the current in the field on the 1-cm scraped portion of the Astato wire. Furthermore, dextrose serves as an electrical conductor, dispersing the electrosurgical current evenly across the tissue to prevent excessive heat concentration at the contact point and reduce the risk of thermal injury to surrounding tissues. It also enhances the efficiency of electrosurgical energy delivery by conducting the electrical current effectively and ensuring that the energy is delivered precisely to the target tissue and limiting damage to the surrounding tissue. Although most dissected iliac arteries are ectatic or aneurysmal, limiting the potential damage of intravascular electrocautery, TEAS should be avoided in smaller vessels. In this study, all cases were performed with bilateral access; however, TEAS was performed through one access site to allow for controlled, even traction of the Astato wire during septotomy. Thus, it might be feasible to perform this procedure via unilateral access. It is important to note that only two of these procedures were performed in a staged fashion with TEAS performed before complex EVAR. Although no patients experienced symptoms of spinal cord ischemia after aortic repair, the use of staged TEAS might allow for increased perfusion of the spinal cord vessels that had previously originated from the false lumen. Thus, in long segment chronic dissecting TAAAs, staged TEAS might allow for preconditioning of these vessels and their collateral vessels to support the second-stage coverage. However, further studies are needed to support this principle. The main challenges we foresee with the use of TEAS are related to the presence of calcium and large amounts of thrombus around the septum. For such cases, TEAS might be contraindicated. Future studies will help to determine the specific contraindications to TEAS and elucidate the true benefits of the procedure before EVAR.

### Study limitations

The limitations of the current study include its retrospective design and small patient population. Furthermore, there is an inherent selection bias in this study that limits the generalizability of the findings to a broader population. However, even with the small patient population, we demonstrated statistically significant expansion of the TL diameter and CSA. Furthermore, this study is constrained in its ability to compare the benefits of TEAS against established surgical methods within the field. Another limitation of this study is its inability to make direct comparisons between operative times and fluoroscopy and radiation data due to differences in the surgical approaches and procedure timing. Specifically, a subset of patients underwent TEAS with concomitant EVAR, TEVAR, or FBEVAR, and another subset underwent staged interventions. Further studies could examine whether there exists a discernible disparity in aortic repair operative times after TEAS compared with alternative procedures. In particular, these studies might identify any differences in the ability to cannulate target vessels during FBEVAR after septotomy. This study focuses on the development of the new surgical technique and evaluating the feasibility and safety of TEAS. It does not adequately evaluate the long-term outcomes or durability of complex EVAR after septotomy. Future directions of research in the treatment of chronic dissecting TAAAs should prioritize comprehensive, long-term comparative studies between TEAS and established methods for addressing a chronic septum to better discern the role of TEAS before complex EVAR.

## Conclusions

TEAS represents a promising approach for the treatment of chronic dissecting aortoiliac aneurysms. Our results support its safety and effectiveness in achieving technical success, expanding the TL, and creating favorable conditions for subsequent EVAR. Further research and long-term follow-up studies are warranted to validate these findings, assess the durability of this innovative technique, and compare it to known surgical techniques.

## Author Contributions

Conception and design: MT, AF, KL, JC, MG, MB, CT

Analysis and interpretation: MT, AF, KL, JC, MG, MB, CT

Data collection: MT, AF, JC, MG

Writing the article: MT, AF, JC, MG, CT

Critical revision of the article: MT, AF, KL, JC, MG, MB, CT

Final approval of the article: MT, AF, KL, JC, MG, MB, CT

Statistical analysis: Not applicable

Obtained funding: Not applicable

Overall responsibility: CT

## Disclosures

M.S.B. and C.H.T. have been consultants for, and received research support from, 10.13039/100010479Cook Medical Inc, W.L. Gore & Associates, Inc, and Phillips Healthcare. The remaining authors report no conflicts.
